# 

**Published:** 1901-11-23

**Authors:** 


### NOTICE TO CORRESPONDENTS.

All MSS., Letters, Books for Review, and other matters intended for the Editor should be addressed THE EDITOR, "THE HOSPITAL," THE HOSPITAL BUILDING, 23 & 29 SOUTHAMPTON STREET, STRAND, W.C.

The Editor cannot undertake to return rejected MS., even when accompanied by stamped directed envelope.

**Notice to Advertisers and Subscribers.**

All Advertisements, Orders for Copies of THE HOSPITAL, and Business Communications should be addressed to **THE MANAGER (Not to the Editor),** THE HOSPITAL, 28 & 29 Southampton Street, Strand, London, W.C.

The Cost of Subscription, post free, is as follows:—Per week, 2½d.; per quarter, 2s. 8d.; per half-year, 5s. 3d.; per annum, 10s. 6d. Foreign:—Per annum, 15s. 2d., payable, in all cases, in advance.

**NOTICE.—Vol. XXX. of "The Hospital" (April 1901, to September 1901), in handsome cloth binding can now be obtained at the office of this Journal, price, 7s. 6d. Cases for binding the Numbers contained in this Volume 1s. 6d. Index to Vol. XXX., 2½d., post free.**

**The Monthly Part for December will be ready on December 2nd. Price 6d., by post 8d.**

### BOOKS RECEIVED.

Sonnenschein and Co.

"What's What." A Guide for To-day to Life as it is and Things as they are. 1902. By Harry Quilter, M.A.

Baillière, Tindall, and Cox.

"Menstruation and its Disorders." By Arthur E. Giles, M.D., B.Sc., F.R.C.S., M.R.C.P.

"On the Cure of the Morphia Habit without Suffering." By Oscar Jennings, M.D. (Paris), M.R.C.S. (Eng.).

"Aids to Obstetrics." By Samuel Nall, B.A., M.B., D.P.H., M.R.C.S.

### SCRAPS AND GLEANINGS.

The four days' bazaar on behalf of the Bradford Children's Hospital has resulted in the raising of a sum of £5,200.

The Committee of Management of the Belgrave Hospital for Children have received £100 from the Mayor of Wandsworth towards the special appeal now being made for the building fund of the new hospital.

The number of in-patients treated during the past year at the Stirling Royal Infirmary amounted to 256, being an increase of 11 on the previous year's return, whilst the number of out-patients was 2,339, being an increase of 228.

It is stated that the total amount realised by the appeal on behalf of the Nottingham Children's Hospital will probably considerably exceed £4,000. On the last day of the bazaar in its aid the takings at the door and at the stalls amounted to £1,253.

### The Student of Medicine.

### APPOINTMENTS.

**Ernest W. Cross**, M.R.C.S.Eng., L.R.C.P.Lond., appointed Medical Officer to the Infant Orphan Asylum, Wanstead. **Patrick Dempsey**, F.R.C.S.I., M.R.C.S., L.R.C.P., appointed Surgeon for Disease of the Throat and Nose to the Mater Misericordiæ Hospital. **A. J. Eades**, L.R.C.P. & S.I., appointed Senior Assistant Medical Officer at the County Asylum, Winwick. **Wallace Eales**, L.R.C.P., L.R C.S., appointed Honorary Surgeon to Hulme Dispensary, Manchester. **Donald Gordon Falconer**, M.B., M.S. Aberd., appointed Certifying Factory Surgeon for the Foyer. District of Inverness-shire. **Edmund Glenny**, L.R.C.P & S.I., appointed Junior House Surgeon to the Jarvis Street Hospital, Dublin. **Clement Hadley**, L.R.C.P.I., M.R.C.S.Eng., appointed Certifying Factory Surgeon for the Shilton District of Warwickshire. **E. L. Hunt**, L.R.C.P. & S.I., appointed District Medical Officer of the Malmesbury Union. **Vincent Howard**, M.R.C.S., L.R.C.P., appointed Certifying Factory Surgeon for the Borough and Rural District of Buckingham. **Thomas Hunt**, M.R.C.S. Eng., L.R.C.P.Edin., appointed Resident Medical Officer, St. George's Retreat, Burgess Hill, Sussex. **H. Johnson**, L.R.C.P., L.R.C.S.Edin., appointed District Medical Officer of the Newark Union. **J. P. Johnstone**, L.R.C.P., L.R.C.S.Edin., appointed Medical Officer of the Langport Union Workhouse. **J. Llewellyn**, M.R.C.S.Eng., appointed Medical Officer for the Sherburn District of the Scarborough Union. **Ernest Colwell Maguire**, M.D., C.M.Aberd., appointed Surgeon to the Brighton Borough Police. **Lawrence O'Clery**, M.D., M.Ch.R.U.I., appointed Medical Officer for the Clonalkity Workhouse. **Frank Ogston**, M.D., M.S.Aberd., appointed District Medical Officer for the Southern Half of the South Island of New Zealand. **T. W. Parry**, M.B., B.C.Camb., appointed Certifying Factory Surgeon for the Youlgreave District of Derbyshire. **R. W. Prentice**, L.S.A., appointed Medical Officer to the Ringwood District Council. **J. S. Prowse**, M.B., B.C.Cantab., appointed Honorary Physician to the Hulme Dispensary, Manchester. **W. J. E. Sumpter**, M.R.C.S.Eng., L.R.C.P.Lond., appointed Medical Officer of Health for the Sheringham Urban District Council. **James Arthur Thompson**, M.B., B.Ch., B.A.O.T.C.D., appointed Senior House Surgeon to the Jervis Street Hospital, Dublin. **Miss E. S. M. Walker**, M.B., B.S. Glasg., appointed Assistant Medical Officer at the Toxteth Park Workhouse.

